# Effects of Residual Stress Distribution on Interfacial Adhesion of Magnetron Sputtered AlN and AlN/Al Nanostructured Coatings on a (100) Silicon Substrate

**DOI:** 10.3390/nano8110896

**Published:** 2018-11-01

**Authors:** Rashid Ali, Marco Renzelli, M. Imran Khan, Marco Sebastiani, Edoardo Bemporad

**Affiliations:** 1 Faculty of Materials and Chemical Engineering, Ghulam Ishaq Khan Institute of Engineering Sciences and Technology, Topi, Swabi 23640, KPK, Pakistan; rashidali@giki.edu.pk (R.A.); imrankhan@giki.edu.pk (M.I.K.); 2 LFoundry s.r.l., via Pacinotti, 7 Avezzano, 67051 L’Aquila, Italy; renzelli.marco@gmail.com; 3 Engineering Department, Università degli studi Roma Tre, via dellaVasca Navale, 79, 00146 Rome, Italy; edoardo.bemporad@uniroma3.it

**Keywords:** physical vapor deposition, magnetron sputtering, AlN/Al coating, silicon substrate, residual stresses, wafer curvature method, nanoscale residual stress profiling, indentation failure modes, nanoindentation adhesion

## Abstract

The present study investigated the influence of nanoscale residual stress depth gradients on the nano-mechanical behavior and adhesion energy of aluminium nitride (AlN) and Al/AlN sputtered thin films on a (100) silicon substrate. By using a focused ion beam (FIB) incremental ring-core method, the residual stress depth gradient was assessed in the films in comparison with standard curvature residual stress measurements. The adhesion energy was then quantified by using a nanoindentation-based model. Results showed that the addition of an aluminum layer gave rise to additional tensile stress at the coating/substrate interface, which can be explained in terms of the differences of thermal expansion coefficients with the silicon substrate. Therefore, the coatings without the Al layer showed better adhesion because of a more homogeneous compressive residual stress in comparison with the coating having the Al layer, even though both groups of coatings were produced under the same bias voltage. Results are discussed, and some general suggestions are made on the correlation between coating/substrate property combinations and the adhesion energy of multilayer stacks. The results suggested that the Al bond layer and inhomogeneous residual stresses negatively affected the adhesion of AlN to a substrate such as silicon.

## 1. Introduction

High-quality thin coatings of aluminium nitride (AlN) have been extensively used in electronics for heat dissipation applications [1]. These coatings have been produced by several established methods. However, due to simplicity, reproducibility, and lower cost, magnetron sputtering remains one of the common techniques used for the deposition of AlN coatings. One of the peculiar features of many physical vapor-deposited (PVD) coatings is the presence of high compressive residual stresses. These stresses are an intrinsic outcome and could be a limitation for the maximum achievable thickness and/or for optimized adhesion to the substrate.

In a recent review article [2], the origin and evolution of residual stress in thin films is discussed in detail and the correlations between process parameters, microstructures, and stress distributions are analyzed. Here, it is shown how complex residual stress depth gradients can be generated during the growth of PVD thin films. Those stresses, even if compressive on average, can have an unwanted effect on the mechanical response of thin films during service. This is also true in case of Atomic Layer Deposition (ALD) dielectric layers (which can be denser and perfectly conformal to the substrate, in comparison with PVD), where the presence of residual stress can generate cracks [2]. Recent papers by some of the authors [3,4] showed that the presence of a controlled residual stress gradient in multilayer Cr–CrN PVD thin films can have a very beneficial effect on scratch adhesion and can be a powerful design tool for nanostructured coatings with improved performance. Therefore, one of the most important avenues of research for magnetron sputtered PVD coatings is to increase the adhesion of the coating to the substrate under optimized residual stress gradients [3,4].

Similar to the commonly used Rockwell-C indentation and scratch testing techniques for determining the adhesion of a thick hard coating on a ductile substrate, nanoindentation is also used as an enabling technique to investigate the adhesion of thin coating systems onto hard substrates. Especially in the case of very thin nanostructured layers (<1 μm), standard scratch and HRC tests fail to output reliable adhesion values, since the response of the coating/substrate system becomes a strong function of the substrate’s properties and may change remarkably as a function of the adopted indenter tip. There are several additional benefits that make nanoindentation attractive; only a small sample is needed to perform the test that can potentially supply several materials properties, such as hardness, modulus, and adhesion [5]. There are few studies in the authors’ knowledge that quantitatively investigate the effect of residual stresses on contact-induced coating adhesion and delamination mechanisms. A quantitative measurement of coating adhesion is essential to allow their exploitation for many critical applications, particularly within the semiconductor microelectronics industry [6,7]. This paper reports the work that aimed to clarify the role of interfacial residual stresses in the adhesion of thin films, as measured by nanoindentation coupled with scanning electron microscopy (SEM) investigations of indentation-induced failure modes.

To this purpose, four different coating systems were produced: a simple AlN single layer on a Si substrate, where the film was expected to be in compression; the same AlN layer with the addition of an aluminum bond layer that was designed to have tensile residual stress in the aluminum layer (as it usually happens for Al metallic sputtered layers), to investigate the effect of an inhomogeneous state of stress (tensile–compressive) on the adhesion of the coating. The same AlN coatings (with and without an Al bond layer) were also deposited under higher bias voltage conditions, in order to investigate the effects of an increased compressive residual stress in the top layer on adhesion. Average residual stress and through-thickness stress gradient were measured for their influence on interfacial adhesion. Through-thickness distribution of the residual stress gradient and mechanical properties are essential for characterizing nanostructured materials [8].

Finally, by combining the results from the four different samples, a discussion was made on how the residual stress distribution in Al/AlN sputtered coatings would affect adhesion and crack propagation modes.

## 2. Experimental Details

Coatings were produced using a standard PVD magnetron sputtering plant, available at Roma Tre University facilities, with direct current (DC)-powered aluminium targets and a radio frequency (RF)-powered capacitive coupled sample holder capable of inducing bias to conductive and dielectric substrates. More details on the deposition geometry are reported in previous papers [3,4]. Nitrogen was put into the chamber to produce aluminium nitride by reactive sputtering. The aluminium nitride thin films were deposited on ≈290 µm thick rectangular (20 mm long and 7 mm wide) single-crystal (100) silicon wafers with an initial average roughness (Ra) of ≈10 nm. Prior to sputtering, silicon substrates were cleaned with acetone and ethanol for five minutes in each. Samples were mounted on a sample holder that was placed at a distance of 80 mm from the target. These substrates were subsequently sputter-etched in argon plasma for 10 min in order to remove the surface oxide layer. Four samples were deposited with an AlN coating using 180 W DC on the 200 mm Al cathode, a gas mixture of 25% argon and 75% nitrogen, a process pressure of 1 × 10^−4^ mbar, and a base pressure lower than 6 × 10^−6^ mbar. With these deposition parameters, the cathode was fully poisoned, ensuring a perfect stoichiometry of the produced coatings; the low power density on the aluminium targets ensured no arcing and thus no particle production. The four samples differed for the applied negative bias voltage to the substrate during growth and the optional presence of a 50 nm thin pure aluminium bond layer; the samples with or without the bond layer were coated with floating potential (around 30 V from the plasma) or 100 V RF applied potential. The maximum temperature developed during deposition and ion etching was measured with a temperature measurement strip gauge. These strip gauges were mounted onto the aluminum sample holder which held the sample on other side. At the end of deposition, a maximum temperature of ≈150 °C was recorded. To facilitate reading, the produced coatings were investigated by dividing into two groups: one without the bond layer under a floating bias of 30 V and bias voltage of 100 V, named AlN-30V and AlN-100V, respectively; in the second group was an AlN coating under a bias voltage of 30 V and 100 V with an aluminium bond layer, named AlN/Al-30V and AlN/Al-100V, respectively.

After deposition, the wafer curvature method (extended Stoney formula) was used for average residual stress measurement, in which the biaxial state of stress was taken into consideration [9,10]. The choice of (100) silicon, with its more complex stiffness with respect to (111) silicon, was due to the need to have perfectly rectangular strips ((111) silicon tends toward triangular cleavage). Curvature profiles were measured on the coating side. As widely known from the literature, a concave surface curvature corresponds to compressive residual stress in the characterized coatings. Curvature was then measured using a state-of-the-art optical profilometer (Leica DCM-3D, Bannockburn, IL, USA) in accordance with the standard procedure CEN/TS 1071-1 [11]. An optical profilometer enables accurate and reproducible curvature measurements with z-height resolution of ≈0.6 nm. A two-dimensional (2D) coating surface profile with up to 8 mm scan length was constructed with extended topography in confocal mode. The radius of curvature was measured through circle fitting of the 2D profile, and residual stresses were calculated by the modified Stoney equation:

(1) $$\;\sigma =M_{\left(100\right)}\frac{t_{s}^{2}}{6t_{c}}\frac{1}{R}$$

where t_s_ and t_c_ are the thickness of the substrate and coating, respectively, and M (100) = $\frac{E_{s}}{1-\vartheta_{s}}$ is the biaxial modulus of single-crystal silicon (100), which is 180.4 GPa [12]. In the equation above, $E_{s}$ and $\vartheta_{s}$ are the elastic modulus and Poisson’s ratio of the substrate, respectively. The thickness of the coating was measured with an optical profilometer (step height method) and verified with focused ion beam (FIB) FEI Helios NanoLab 600 Dualbeam FIB/SEM (Thermo Scientific, Hillsboro, OR, USA) cross-section analysis.

The through-thickness gradient of residual stress was measured by using a novel incremental micro-ring-core profiling method [13,14,15], which consists of controlled material removal by focused ion beam (FIB) microscopy, coupled with SEM high-resolution imaging, digital image correlation (DIC) for relaxation strain analysis, and finite-element (FEM) calculation of the residual stress depth gradient. As described in a recent paper, this new method allows for nanoscale depth profiling [15] of the residual stress in thin films. In this paper, this method was applied to the coatings with an Al interlayer (both deposited at −30 and −100 V bias voltage) in order to investigate the residual stress gradient in proximity of the AlN/Al interface. The same method also allowed for the estimation of the average residual stress in the film, and a comparison with the results from curvature analysis was made.

The elastic modulus and hardness of coatings were measured by nanoindentation testing in accordance with the ISO 14577 standard (Keysight G200 Nano Indenter^®^, 0.05 s^−1^ constant strain rate, maximum indentation depth 1000 nm) by employing the continuous stiffness method (CSM), which allows to calculate the hardness (H) and elastic modulus (E) profiles versus penetration depth (it is the superposition of a oscillating load to the linear increment in the indenter depth). For all nanoindentation experiments, the frame compliance and indenter tip area function were calibrated on a standard fused silica block and following the procedure suggested by Oliver and Pharr [16]. As the coating had a thickness in the order of ≈700 nm, the most reliable values of hardness and modulus were obtained by interpolation of the CSM hardness/modulus versus depth profiles and the calculation of the interpolated average H and E values in the depth range of 60–70 nm. In this way, the effects coming from the substrate’s influences are reduced (this is especially true for hardness, while the elastic modulus is always influenced by the substrate compliance).

Adhesion of coatings was assessed by equipping the nanoindenter with a cube-corner tip and making load-controlled indentations in order to induce coating delamination, using a maximum load of 200 mN. Nine indents on each sample were made and at least five indents were observed by scanning electron microscopy (SEM). The interfacial adhesion of coatings under different residual stress conditions were evaluated using the model described by den-Toonder et al. [17], through microscale observation of indentation-induced failures. Originally, Thouless [18] proposed this model for interfacial adhesion through the observation of triangular coating spallation failures with scratch tests. Later, den-Toonder et al. [17] modified this model to take into account the curved geometry of the delaminated segment and residual stresses, which usually produces failures in residual stressed coatings under nanoindentation. In this work, this model was used to quantify the interfacial adhesion of the films.

## 3. Results and Discussion

### 3.1. Residual Stress Measurement

**Figure 1:**
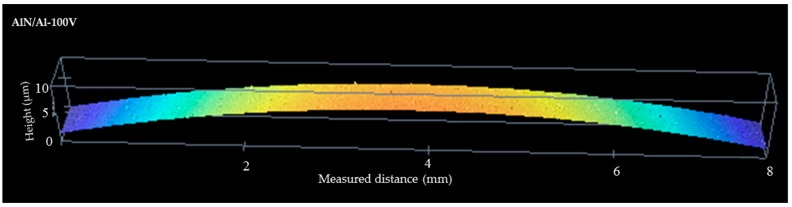
Example of optical profilometer-measured radius of curvature of the coatings after deposition.

An isometric view of a typical 2D profile of silicon wafer curvature measured with an optical profilometer is shown in Figure 1; curvatures for other samples are not shown here in the interest of simplicity.

**Figure 2:**
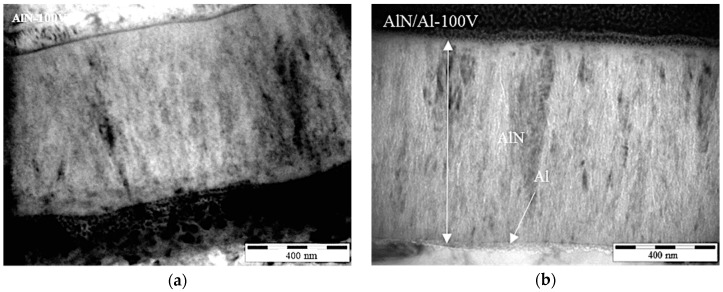
TEM observations (of focused ion beam cross sections) of the produced coatings for coating thickness measurement: (**a**) AlN-100V; (**b**) AlN/Al-100V.

Coating thickness and microstructure were analyzed with FIB and transmission Electron Microscopy (TEM) section analysis (Figure 2), while residual stress was calculated using the curvature method, as reported in Table 1. As clearly visible in Figure 2, the presence of an Al bond layer does not change the microstructure of the AlN layers, which are made of very fine columnar nanostructured grains.

**Figure 3:**
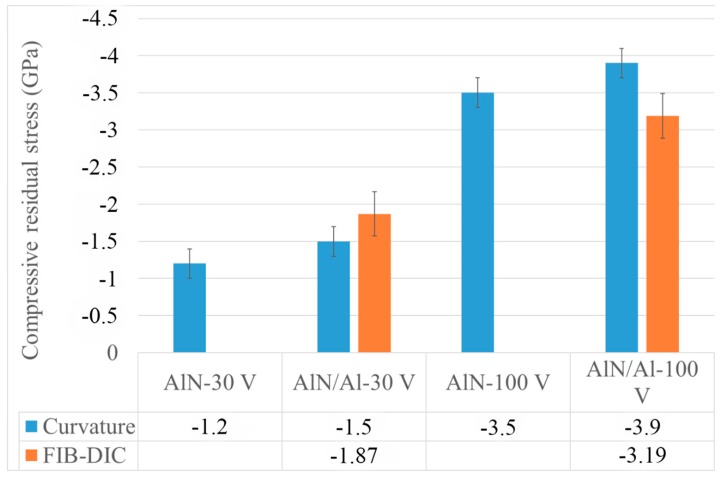
Plot of average compressive residual stress (curvature measurements) and focused ion beam–digital image correlation (FIB-DIC) in all the produced coatings.

The average residual stress values are shown in Figure 3, as calculated by Stoney and FIB-DIC methods. For the two samples where two techniques are used, there is good agreement between the data (i.e., the discrepancy is within the standard deviations), as also reported in previous literature.

On comparing the coatings in the first and second groups, significantly higher residual stress was found under polarization of the substrate with negative potential of 100 V (AlN-100V coatings), compared to the substrate under polarization of 30 V (AlN-30V coating). This is an expected result, and the increase of the average compressive residual stress for all coatings that were deposited at −100 V bias voltage can be attributed to an atom peening effect given by the increased energy of the vapor flux. In case of the AlN coatings on silicon, the thermal component of the residual stress can be considered as negligible with respect to the atomic peening component, since the thermal expansion coefficients of the two materials (AlN and silicon) are similar.

On the other hand, in the AlN coating with an Al bond layer, the thermal stress component has contributed significantly to an increase in the overall residual stress in the AlN/Al coating system, as shown in Figure 3 (under the assumption that the atomic peening component remains the same). Castanho et al. [19] observed a similar phenomenon in magnetron sputtered multilayer coatings, whereas the number of aluminum interlayers was increased, more compressive stresses were induced in the top layer of AlTiN. In fact, the contribution of thermal stresses was analytically evaluated to be ≈+340 MPa by using the thermal expansion of the substrate (Si, 2.3 × 10^−6^ °C^−1^) and bond layer (Al, 22.8 × 10^−6^ °C^−1^) reported in the literature [7]. The aluminum bond layer during cooling from the deposition temperature attempted to contract more than the silicon substrate and the AlN coating. However, at the interfaces, the aluminum cannot contract fully due to being constrained by the silicon substrate and AlN top layer, and as a result, the aluminum bond layer is in tension, which causes additional compression residual stresses in the AlN coating and silicon substrate. By taking these thermal stress contributions into account, the residual stress variation between the coatings with and without the bond layer as reported in Figure 3 is largely explained.

Furthermore, the FIB-DIC analysis of residual stress depth profiles in the AlN layers is a further confirmation of the above discussions. In fact, in both cases (AlN/Al with −30 V and −100 V bias), we do observe a strong surface compressive residual stress that goes towards tensile when approaching the AlN/Al interface. In the case of the AlN/Al-100V system, we even observed a mild state of tensile stress in the AlN layer in the vicinity of the interface. For this sample, the presence of tensile stress when approaching the interface is a further explanation for the extensive delamination that was observed during the indentation.

**Figure 4:**
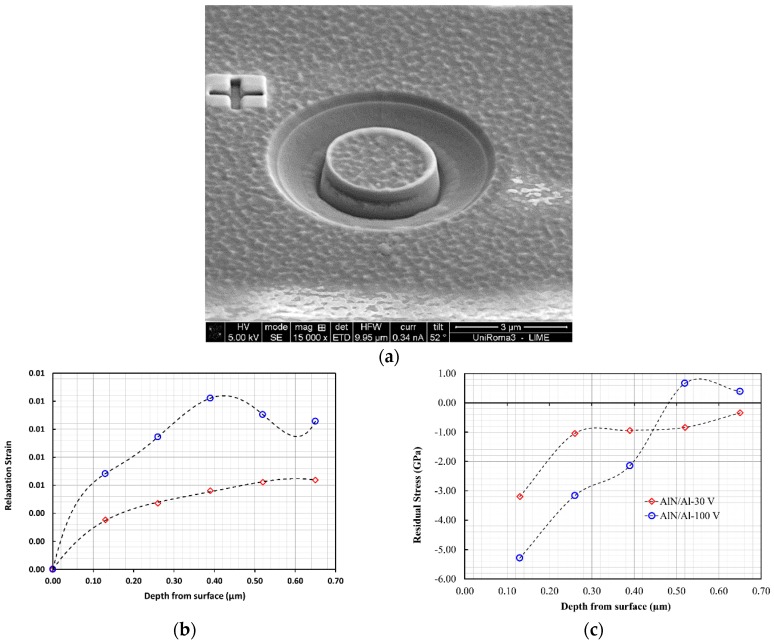
Results of FIB-DIC residual stress characterizations. (**a**) Example of the micro-ring-core test; (**b**) Representative relaxation strains vs. milling depth for the two coatings with the Al bond layer; (**c**) Representative residual stress profiles for the two coatings with the Al bond layer (AlN/Al with −30 V and −100 V bias), obtained by using the FIB-DIC depth profiling method described in [15].

Therefore, the adoption of an additional Al interlayer, in this specific case, gave rise to an increased compressive stress in the top layer (AlN), and at the same time, a stronger stress gradient and an additional tensile residual stress at the interface (Figure 4c).

### 3.2. Nanoindentation Characterizations

#### 3.2.1. Hardness and Elastic Modulus

**Figure 5:**
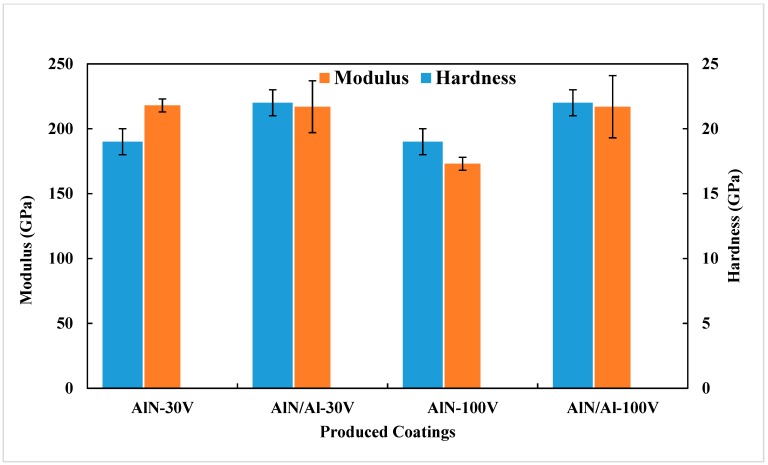
Results of nanoindentation hardness and elastic modulus.

The hardness and elastic modulus of all the produced coatings were measured with the Berkovich indenter, and results are summarized in Figure 5.

**Figure 6:**
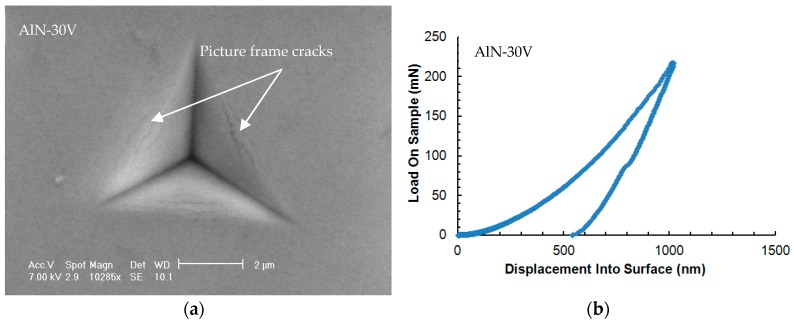

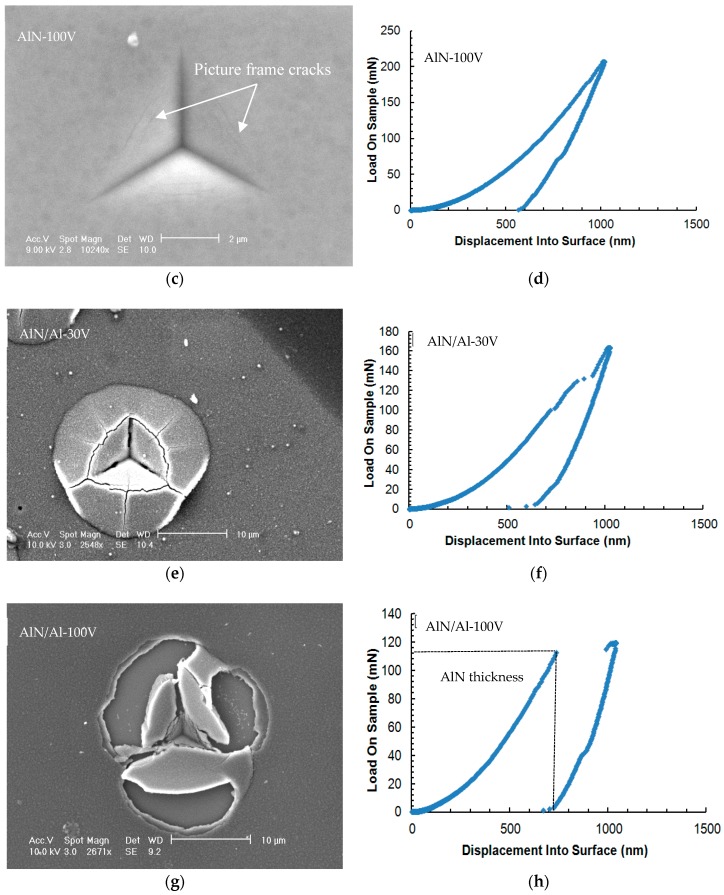
The SEM micrographs of indentations (**left**) made with the Berkovich indenter and the associated load–displacement curves (**right**). (**a,b**) AlN-30V, (**c,d**) AlN-100V, (**e,f**) AlN/Al-30V, (**g,h**) AlN/Al-100V.

There is a minor difference in the measurements between AlN coatings with and without the bond layer. Coatings made with the bond layer have higher standard deviations that those without it. The major source of deviation in nanoindentation hardness and modulus is roughness of the coating surface, and it is possible to see from Figure 6 that the surfaces of the coatings AlN-30V and AlN/Al-30V are different. 

The nanoindentation results further demonstrate that the only (and major) difference among the coatings is the residual stress and its distribution. Therefore, the main difference in adhesion could only be attributed to the different residual stress distribution.

The subsequent sections summarize the results of coating/substrate interfacial adhesion, failure modes, and their effect analysis. 

#### 3.2.2. Quantitative Evaluation of Coatings’ Interfacial Adhesion

The interfacial adhesion energy of the coatings was investigated by analyzing microscale delamination failures produced under Berkovich and cube-corner indenter tips. At first, nanoindentations were made with standard Berkovich indentation tip. Figure 5 shows the load–displacement curves and the associated SEM micrograph of an indent in the four cases under consideration.

As clearly visible from Figure 6, the coating/substrate adhesion decreases dramatically for the two coatings with an Al layer.

The discontinuous cracks formed in the indents of the AlN-30V and AlN-100V coatings (as indicated by the picture-frame cracks shown in Figure 6) seem to be due to tensile stresses induced by the dragging of the part of the coating around the indenter from the bulk of the coatings. An extensive analysis of this kind of cracks is reported in [20], where it is explained that they are related to the E/H (elastic modulus to hardness) ratio mismatch between the coating and substrate. The discontinuity in displacements during the unloading segment is related to the reverse phase transformation in the silicon substrate [21].

Only the coatings with the Al bond layer, AlN/Al-30V and AlN/Al-100V, presented extensive buckling and delamination with the Berkovich indenter at 140 mN and 120 mN indentation loads, respectively, as shown in the right column of Figure 5. The load–displacement curve in the coating AlN/Al-100V at the thickness of ≈750 nm exhibited a displacement burst (horizontal step) and confirms the through-thickness cracking leads to the interfacial detachment of the coating. The metallic film (bond layer) is likely to undergo plastic deformation and these deformations could be significantly larger than the film thickness [22]. From these observations, it was clear that the coating AlN/Al-100V has the poorest adhesion, as it delaminated completely at the lower load and it also had higher residual stress in comparison with the AlN/Al-30V coating.

**Figure 7:**
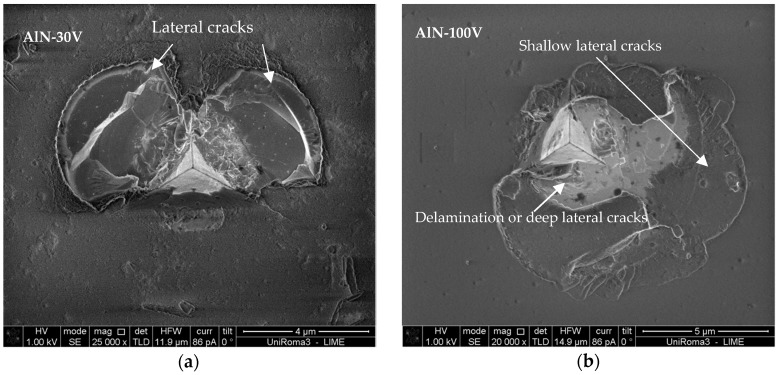
The SEM micrographs of indentations made with a cube-corner indenter on the coatings: (**a**) AlN-30V and (**b**) AlN-100V (at different magnifications).

After these tests, all the four coatings were tested again with a cube corner indenter [23] to produce fractures. The coatings (AlN-30V and AlN-100V) without the bond layer did not delaminate up to the substrate (Figure 7) and only showed cracks within the film thickness.

The SEM indentation micrographs of the coatings AlN-30V and AlN-100V clearly shows that the central region is shallow to various depths compared to the mean level of the coating (Figure 7) and the cracks’ morphology is similar to the usually observed lateral cracks [24]. The propagation of cracks through the formation of steps, followed by turning upward to form chips, is confirmation of lateral cracks, and crack propagation is similar in the two coatings’ micrographs. It is possible that higher residual stress magnitude caused the chipping (possible delamination) failure, seemingly due to weaker adhesion in the AlN-100V coating. However, for the AlN-30V coating, having good adhesion, the indenter caused lateral cracks without delamination. The possibility of change in the crack pattern with a sharp cube-corner indenter is expected as a result of the substrate effect. 

For this lateral cracking in the coatings AlN-30V and AlN-100V, a fracture toughness value of 1.8 and 2.3 MPa·m^0.5^ was estimated with the den-Toonder model (Equation (1)) [17], which was originally developed by Antis et al. [25] to investigate the fracture toughness of bulk materials. The inputs of this model are the crack load (80 and 100 mN for the coatings AlN-30V and AlN-100V, respectively), the length of lateral cracks, the crack depth which is equal to half the coating thickness, and the measured residual stresses in the coatings. It has to be underlined that such values should be only considered as providing a rough estimation of a coating’s fracture toughness, since there are many factors affecting the reliability of such indentation-based methods, as discussed in recent papers [26]. Nonetheless, the estimated fracture toughness values of the coatings are very similar and in the range of the toughness (1.5–5 MPa·m^0.5^) of ceramic coatings reported in the literature. This estimation has an error of 0.2 MPa·m^0.5^ (normally, the error is much higher for this kind of measurement, at around 30%); this low error value comes from the repeatability of the results. It was expected that higher stresses under higher bias voltages were to be correlated with higher toughness of the coating, but this was not the case. The measurements show that the higher load needed to crack the coating AlN-100V is due to the compressive intrinsic stresses, and once this effect is taken into account, there are no major toughness difference between the two coatings.

**Figure 8:**
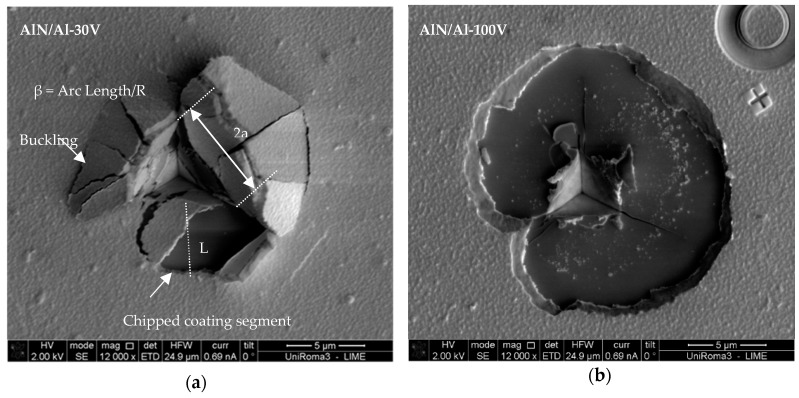
The SEM micrographs of indentation-induced failures for adhesion measurement of (**a**) AlN/Al-30V and (**b**) AlN/Al-100V coatings.

For the coatings with the Al bond layer, the cube-corner indenter achieved full coating delamination from the substrate, and the den-Toonder model [17] could be used to quantify the interfacial adhesion energy. According to this model, the indenter only has the role of crack initiation, and residual stresses are the driving force for delamination and chipping. The inputs of this model are the measured coating thickness, elastic modulus, residual stresses, and chipped coating segment geometric information, as elaborated in Figure 8a.

**Figure 9:**
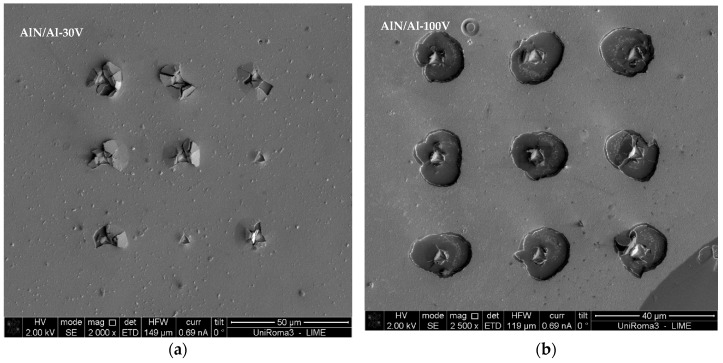
The SEM micrographs of indentation-induced failures of all the indents made for adhesion measurement in (**a**) AlN/Al-30V and (**b**) AlN/Al-100V coatings (at different magnifications).

Coating segments formed chips, which can be clearly seen in the micrograph of the AlN/Al-30V coating, in a semicircle with an easily identifiable radius, and the chipped region has similar schematics for which den-Toonder developed the model (Figure 8). With comparison of the microscale delamination failure mode (semicircular) in Figure 7, it is also possible to see that the circular chipping in the AlN/Al-100V coating was larger than that in the AlN/Al-30V coating. The AlN/Al-30V coating had lower residual stress in comparison with the AlN/Al-100V coating, and having higher residual stresses resulted in different delamination failures; however, the mode of delamination in almost all of the indents was circular (Figure 9).

The model of den Toonder et al. [17] (Equation (10)) resulted in the interfacial adhesion energy of 5 and 4 J/m^2^ for the AlN/Al-30V and AlN/Al-100V coatings, respectively. The estimations have an error of ±5%. The calculated adhesion energy is the practical interfacial fracture energy and it also includes the elastic strain energy associated with the release of residual stresses. The interfacial fracture energy or the adhesion energy results are in accordance with the qualitative comparison of indentation-induced failures produced with the cube-corner indenter. In addition, the adhesive failure modes of the AlN/Al-30V and AlN/Al-100V coatings were compared with the literature, and were in good agreement with those given by Bull [27]. The results indicate that the adhesion of the coating has been deteriorated, in this specific case, due to the presence of the aluminum bond layer. As the AlN-30V and AlN/Al-30V coatings have a difference in compressive residual stress of only +340 MPa, we can expect that the Al bond layer is in a tensile stress state with a similar magnitude.

This assumption can be justified by considering the additive nature of residual strains in multilayer systems. The bond layer, being ductile, cannot store elastic energy across the interface, and tensile residual stresses are also present in it (as also observed by FIB-DIC stress profiles). The tension coupled with deformation during indentation in the Al bond layer leads to interfacial delamination. In most of the studies available in the literature, AlN was directly coated onto a silicon substrate; nonetheless, there are a few studies that used a thin layer of Al to improve the bonding between the AlN and Si substrate, even though the adhesion was not investigated [28].

In this work, we have shown that the role of the residual stress in the bond layer is significant, because even a ≈50 nm thin layer is capable of causing a break by bulging the ≈700 nm thick upper layer, decreasing the functional adhesion of the coating under nanoindentation (Figure 9). Therefore, we demonstrate here that the residual stress state in the Al bond layer can have a primary and relevant effect on the coating’s adhesion, more than the residual stress and hardness of the AlN top layer.

**Figure 10:**
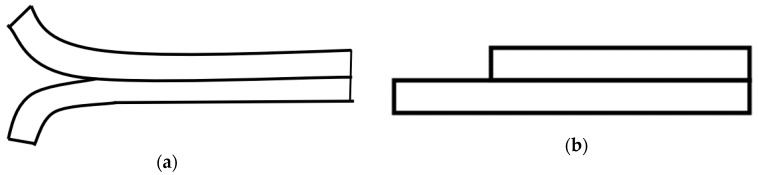
Schematic of interfacial crack initiation modes: (**a**) mode I failure; (**b**) mode II failure.

Also, with bond layer being ductile, one would expect an increase of the interfacial adhesion energy because of the decrease of stored elastic energy across the interface. However, as the indentation depth exceeds the top layer’s thickness (AlN), a step in the load–displacement curve occurs (see right column of Figure 6) in the AlN/Al-30V and AlN/Al-100V coatings. As a result, soft bond layers, such as of pure aluminum and copper, are not adequate to be a proper coating base, especially if the substrate is hard. This is the reason why only bond layers made of Ti, Cr, Mo, or Nb are effective on the hard substrate for the smooth transition of properties between the hard coating and substrate [29,30]. Comparison of coating delamination behavior under different residual stress conditions suggested (Figure 8) that both the mode I and mode II load components in the buckled and unbuckled states are operative. The schematic of both the buckled and unbuckled failure modes are given in Figure 10 for reference. 

**Figure 11:**
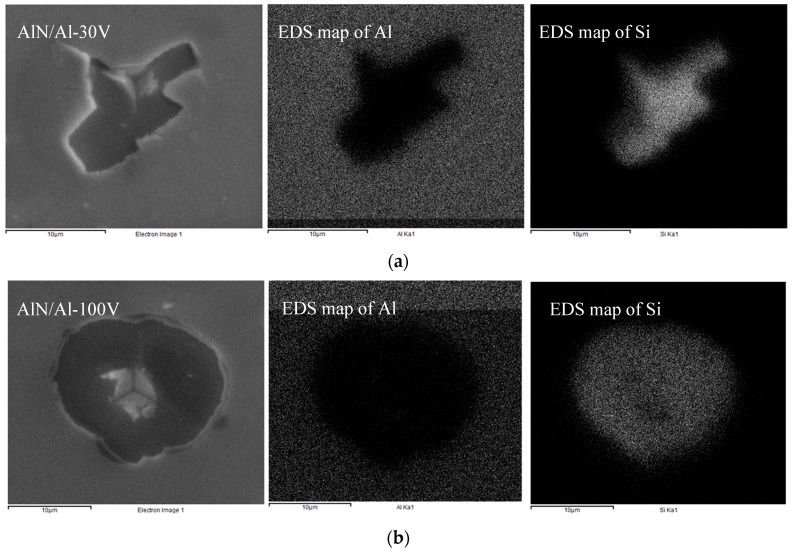
The energy dispersive spectroscopy (EDS) maps of cube-corner produced indentation marks in coatings and the corresponding mapping images of the Al and Si elements: (**a**) AlN/Al-30V; (**b**) AlN/Al-100V.

It can be seen in the micrograph of Figure 8b that the AlN/Al-100V coating shows shear mode II and the failure can be characterized as catastrophic with the highest delamination; however, in the AlN/Al-30V coating, a mixture of peeling (mode I) and shear (mode II) delamination failures were observed as a result of tensile stress in the aluminum coating layer and compressive stresses in the AlN layer. It is possible that at about the critical indenter penetration, the higher compressive stresses of indentation resulted in the buckling of the soft thin film of aluminum, which means that the interfacial adhesion was not too high. The buckling failure is more likely than the shear-induced delamination when the coating is relatively tough compared to the interface. In order to confirm whether the coating delaminates from the AlN/bond layer interface or from the bond layer/substrate interface, energy dispersive spectroscopy (EDS) composition maps were constructed (Figure 11).

The EDS map of aluminum in the second column of Figure 11 shows that aluminum is present in the AlN layer; however, it is not present where the coating has been delaminated. Similarly, the silicon EDS map in the third column from the left shows that silicon is not present in the AlN coating and silicon substrate is exposed after delamination. It is noticeable that silicon substrate is visible and the bond layer is missing in the EDS map, even though the bond layer thickness was measured and confirmed with FIB-TEM cross-section analysis (shown and indicated in Figure 2). This shows that delamination failures are from the bond layer and substrate interface, while the bonding between AlN and Al is perfect. The results indicate that the adhesion of coating has been deteriorated due to the aluminum bond layer and the surface fracture is attributable to the interface between the aluminum and silicon. Using this information and the measured interfacial delamination energy (*G_int_*) of 5 and 4 J/m^2^ for the AlN/Al-30V and AlN/Al-100V coatings, respectively, the interface fracture toughness (*K_int_*) defined by Suo and Hutchinson [31] may be estimated using Equation (2).

(2) $$K_{int}=\;\sqrt{G_{int}E_{int}}$$

where *E_int_* is the interface’s elastic modulus, given by Equation (3).

(3) $$\frac{1}{E_{int}}=\;\frac{1}{2}\;\left[\frac{1}{E}+\frac{1}{E_{s}}\right]$$

Using *E* as the coating modulus and *E_s_* as the substrate modulus, the interface elastic modulus (*E_int_*) was found to be 100 GPa. The estimated interface fracture toughness of 0.71 and 0.65 MPa·m^0.5^ for the AlN/Al-30V and AlN/Al-100V coatings, respectively, clearly indicate why these coatings delaminated instead of cracking, as the resulting interfacial fracture toughness is significantly lower than the cohesive fracture toughness of the AlN-30V and AlN-100V coatings.

There are technological implications of this study’s results for the design and production of coatings on hard substrates, in particular on silicon substrates, for various applications. For example, aluminum nitride and other ceramic coatings are of industrial interest as next-generation materials for sensor applications. Ionescu et al. [32] indicated the real possibilities of manufacturing sensors embedded on the monitored components of machine tools [33]. The strength of silicon wafers used in solar cell and microelectronics applications could be improved with hard coatings [34]. Also, the microscale-produced failure modes and their effect analysis is additive to contact-induced coating failures, which could help to validate the coating adhesion models. In this context, the experimental characterization adds new information to the literature that may help to interpret the contact-induced failures in coatings under tribological situations, by focusing in particular on the role of the residual stress distributions for the case of metal/ceramic multilayer systems.

## 4. Conclusions

The effects of residual stress distribution on the interfacial adhesion energy of coatings were quantitatively investigated and related to the observed micromechanical behavior. The following conclusions could be drawn from this work:

1. An increase in the average compressive residual stress in AlN coatings on the silicon substrate was measured with an increase in bias voltage and coatings deposited with the Al bond layer. Higher bias voltage accelerates the gas ions toward the growing coating and the additional energy caused the enhancement of residual stresses. It is also possible that the different bias conditions affect the interface between Si and AlN, in addition to increasing the compressive residual stresses. The residual stress was further increased by adding an Al bond layer, as a result of its high thermal expansion coefficient in comparison with AlN and the silicon substrate. As a consequence, tensile thermal stresses during cooling were established in the Al, which caused additional compressive residual stresses in the AlN/Al coatings.
2. FIB-DIC residual stress depth profiles showed that for the AlN/Al coatings, a strong residual stress gradient was present in the films, being the maximum compressive residual stress on the top surface, then decreasing towards the interface; a mild tensile stress was observed even for the AlN/Al-100V sample, which was the one showing more extended delamination.
3. The coatings deposited without the Al bond layer showed significantly better adhesion in comparison to those with the bond layer, even though both groups of coatings had differences in compressive residual stresses, equivalent to thermal stresses in the bond layer. Adhesive failures could not be produced in the coatings without a bond layer (AlN-30V and AlN-100V), even with a cube-corner indenter, which means that the interface adhesion was higher in comparison with the coatings deposited with the Al bond layer. The fracture toughness corresponding to lateral cracks was measured to be 2.3 and 1.8 MPa m^0.5^ for the coatings AlN-30V and AlN-100V, having residual stresses of 1.2 and 3.5 GPa, respectively. The observed behavior could be related to more homogenous compressive residual stress distribution in the AlN top layer.
4. The adhesion energy for interfacial separation was found to be 5 and 4 J/m^2^ for the coatings AlN/Al-30V and AlN/Al-100V, having an average residual stress of 1.5 and 3.9 GPa, respectively, with a corresponding interfacial fracture toughness of 0.71 and 0.65 MPa·m^0.5^, which was significantly lower than the estimated fracture toughness of the AlN material, thus explaining why delamination occurs for AlN/Al systems. The tensile residual stress on the bond layer, coupled with its plastic deformation during indentation, leads to interface delamination.
5. These results suggested that in order to be beneficial for adhesion, a metallic bond layer should possess good hardness, a small difference in the thermal expansion coefficient, and a comparable elastic modulus with both the substrate and top coating. Refractory metals are a good prospect for the design of improved multilayer coating systems with optimal performance.

## Figures and Tables

**Table 1:** Description of coatings’ deposition parameters, measured overall coating thickness, and compressive residual stress with the wafer curvature method.

Description of Coatings	Bias Voltage (V)	Bond Layer (Yes/No)	Thickness (μm)	Average Residual Stress (GPa)
AlN-30V	−30	No	0.75 ± 0.05	−1.2 ± 0.2
AlN/Al-30V	−30	Yes	0.76 ± 0.02	−1.5 ± 0.2
AlN-100V	−100	No	0.72 ± 0.03	−3.5 ± 0.2
AlN/Al-100V	−100	Yes	0.71 ± 0.04	−3.9 ± 0.2
